# A Spray-Dried, Co-Processed Rice Starch as a Multifunctional Excipient for Direct Compression

**DOI:** 10.3390/pharmaceutics12060518

**Published:** 2020-06-06

**Authors:** Karnkamol Trisopon, Nisit Kittipongpatana, Ornanong Suwannapakul Kittipongpatana

**Affiliations:** 1 Department of Pharmaceutical Sciences, Faculty of Pharmacy, Chiang Mai University, Chiang Mai 50200, Thailand; mickey_imm@hotmail.com (K.T.); nisitk@gmail.com (N.K.); 2 Research Center for Development of Local Lanna Rice and Rice Products, Chiang Mai University, Chiang Mai 50200, Thailand

**Keywords:** co-processing, direct compression excipient, rice starch, spray drying, sodium silicate, cross-linked carboxymethyl starch

## Abstract

A new co-processed, rice starch-based excipient (CS) was developed via a spray-drying technique. Native rice starch (RS) was suspended in aqueous solutions of 10%–15% cross-linked carboxymethyl rice starch (CCMS) and 0.5%–6.75% silicon dioxide (in the form of sodium silicate), before spray drying. The resulting CSs were obtained as spherical agglomerates, with improved flowability. The compressibility study revealed an improved plastic deformation profile of RS, leading to better compaction and tensile strength. The presence of CCMS also ensured a rapid disintegration of the compressed tablets. CS-CCMS:SiO_2_ (10:2.7), prepared with 10% CCMS, 2.7% silicon dioxide, and 40% solid content, was found to exhibit the best characteristics. Compared to the two commercial DC excipients, Prosolv^®^ and Tablettose^®^, the flow property of CS-CCMS:SiO_2_ (10:2.7) was not significantly different, while the tensile strength was 23%: lower than that of Prosolv^®^ but 4 times higher than that of Tablettose^®^ at 196 MPa compression force. The disintegration time of CS-CCMS:SiO_2_ (10:2.7) tablet (28 s) was practically identical to that of Tablettose^®^ tablet (26 s) and far superior to that of Prosolv^®^ tablet (>30 min). These results show that CSs could potentially be employed as a multifunctional excipient for the manufacturing of commercial tablets by DC.

## 1. Introduction

Recent advancements in tableting technology have led to a significant improvement in the production efficacy of pharmaceutical tablets. However, direct compression (DC) of tablets with high-speed tableting machine limits the use of most conventional excipients, as the process is highly influenced by powder characteristics [1]. DC requires the use of excipients with adequate flowability, good compressibility, high dilution potential, and narrow size distribution to ensure the success of tablet production [2]. This has created challenges for the development of new DC excipients.

Starch has been widely used as pharmaceutical excipients as it offers various applications in tableting [3]. In the case of native rice starch (RS), it exhibits small particle size (2–7 um) with polyhedral shape [4] that promotes compressibility and alleviates lubricant sensitivity. However, the small granule size also results in limited flowability [5,6], preventing it from being a logical DC excipient. To circumvent this problem, a modification was carried out to improve the properties and, thus, the functionality.

The regulatory approval of new chemical excipients generally requires safety and toxicity studies, which are time-consuming and costly processes [7]. Co-processing offers an efficient way to overcome this problem via the combining of two or more approved excipients using physical methods. The process synergizes the functionality and/or eliminates undesirable properties of individual excipients, while still preserving their chemical structure and stability [1]. Moreover, the co-processed excipients facilitate tablet production by eliminating the wet granulation process and the use of various excipients. They also promote homogenous flowing of compactible excipients, which make them superior to the physical mixture formulation [8]. This technique affects the characteristics of excipients at the particle level, such as shape, size, size distribution, surface morphology, and porosity. Examples of techniques employed in the preparation of co-processed excipients include dry granulation, wet granulation, hot-melt extrusion, spray drying, freeze-thawing, solvent evaporation, and co-precipitation [9].

Co-processing is usually conducted on plastic and brittle materials to enhance excipient functionality [10]. The viscoelasticity of materials restricts their binding property due to the storage of large elastic energy after compaction [11]. The presence of a brittle material reduces the excess elastic energy during the compression, which prevents the capping and lamination of the tablets [12]. A study on the mechanical properties of MCC and SMCC tablets [13] shows that the addition of silicon dioxide improves the tensile strength of the SMCC tablet through the effect of silicon dioxide on the particle size and particle size distribution of MCC particles [14]. Brittleness of silicon dioxide also reduces the adverse effect of lubricant on the plastic material by the competitive interaction between silicon dioxide and lubricant at the adhesion sites [15,16].

Spray drying is a commonly used co-processing technique in the development of new pharmaceutical excipients, as it provides agglomerate or spherical particles with a narrow size distribution, thus promoting the flowability of materials. This technique is easy to reproduce and allows good control of particle size [9]. A study has been reported on the production of spray-dried rice starch (Era-tab^®^) as a filler-binder for DC. It is presented as a spherical shape agglomerate that exhibits superior flowability to microcrystalline cellulose, lactose, Starch1500^®^, and dibasic calcium phosphate [17]. The results are in agreement with a study conducted on the co-processing of cellulose and silicon dioxide, in which it is shown that the spray drying technique provides a smoother surface and narrower size distribution of material than other co-processed techniques [18].

In the present study, a new rice starch-based, free-flowing excipient, with good binding and disintegrating properties was developed. RS was used as the core, with the binding/disintegrating cross-linked carboxymethyl rice starch (CCMS) and the flow-enhancing silicon dioxide combined via spray drying to create a co-processed particle. The functionality of the resulting co-processed starch as a multi-functional excipient for DC of the tablet was evaluated, in comparison with commercial DC excipients.

## 2. Materials and Methods

### 2.1. Materials

RS was obtained from Thai Flour Industry Co., Ltd (Bangkok, Thailand). Monochloroacetic acid (MCA, CAS No. 79-11-8, Product Code 8004121000) and sodium silicate (CAS No. 1344-09-8, Product Code 1056212500) were supplied by Merck (Hohenbrunn, Germany). Epichlorohydrin (ECH, CAS No. 106-89-8, Product Code E1055) was a product of Sigma–Aldrich (Steinheim, Germany). Silicified microcrystalline cellulose was purchased from JRS Pharma (Product code P9D8L19, Rosenberg, Germany). Spray-dried lactose was the product of Meggle Pharma (Wasserburg, Germany). All other chemicals used were of AR grade or equivalent. Carboxymethyl rice starch cross-linked with ECH (CCMS) was prepared, as reported in our previous study [19]. The ratios of etherification reaction time to cross-linking reaction time was 1:0.67. This synthesis condition was selected based on binding and disintegration properties of CCMS.

### 2.2. Preparation of a Rice Starch-Based, Co-Processed Excipient (CSs) Using a Spray Drying Technique

#### 2.2.1. Selection of an Optimal Spray Drying Condition

A suspension consisting of CCMS, RS, and silicon dioxide was prepared in purified water to contain 20% solid content. In brief, CCMS powder was dispersed in water with continuous stirring until completely swelled. RS was then added into the mixture, followed by silicon dioxide in the form of sodium silicate solution. The mixture was homogenously blended and allowed to stand at room temperature for 12 h. The suspension mixture was fed through an atomizer of a B-290 mini spray dryer (Buchi, Flawil, Switzerland) to generate small droplets of preliminary co-processed rice starches (PCSs) using three different nozzle tip sizes (0.7, 1.4, and 2.0 mm). The inlet temperature, aspirator percentage, and pump percentage were set at 140–190 °C, 80%–95%, and 15%–20%, respectively. The particle morphology was evaluated, and the nozzle tip size that yielded agglomerate spherical particles was selected. After that, PCSs were prepared at solid content levels of 20%, 30%, and 40% by using the selected nozzle tip. The spray dry parameters were set at 190 °C, 95% of the aspirator, and 20% of the feed pump, respectively. The properties of PCSs were determined, including particle morphology, particle distribution, and flowability. The spray drying condition that provided optimal particles was selected to produce co-processed rice starches (CSs).

#### 2.2.2. CSs Preparation

CSs were prepared using the selected spray drying condition obtained from Section 2.2.1. The co-processed suspension was prepared with different concentrations of CCMS (10%, 12%, 15%) and silicon dioxide (0.5%, 1.0%, 2.7%, 4.05%, and 6.75%) based on dry rice starch weight. The control samples, including spray-dried rice starch (RS-SP), co-processed RS with CCMS (RS-CCMS), co-processed RS with silicon dioxide (RS-SS), co-processed silicon dioxide with CCMS (SS-CCMS) were produced using the same spray dry condition. The physicochemical and pharmaceutical properties of the CSs were investigated and compared with those of RS, RS-SP, RS-CCMS, RS-SS, SS-CCMS, and commercial DC excipients (Prosolv^®^ and Tablettose^®^).

### 2.3. Physicochemical Evaluation

#### 2.3.1. FT-IR Spectroscopy

A Thermo Nicolet Nexus 470 IR spectrophotometer (Thermo Fisher, Waltham, MA, USA) was used to confirm carboxymethyl substitution on CCMS molecules, and to detect chemical interaction among components of co-processed starches. FT-IR spectra of RS, CCMS, spray-dried sodium silicate (SS-SP), and CSs were obtained using θ with KBr before IR recording. The FT-IR spectra of the CSs were compared with those of individual excipients.

#### 2.3.2. X-Ray Diffraction (XRD)

The XRD patterns of starch samples were recorded using an X’Pert-MPD diffractometer (Malvern Panalytical, Malvern, UK) with a reflection mode to determine the crystalline structure of co-processed starches. The diffractogram was registered at Bragg angle (2θ) = 5-40 ° at a scan rate of 2.5 ° per min.

#### 2.3.3. Solubility, Swelling Property, and pH

Swelling power and solubility studies were modified from Wongsagonsup, et al. [20]. Approximately 0.1 g of starch sample powder was accurately weighed into a pre-weighed centrifuge tube and dispersed in 10 mL of purified water (1% w/v). The tube was mixed for 1 min using a vortex mixer and allowed to stand at room temperature for 12 h. The pH of starch suspension was measured, and the suspension was then centrifuged at 2200 rpm for 15 min. The clear supernatant was transferred to a pre-weighed crucible and dried at 110 °C for 24 h. The weight of swollen starch sediment and dry weight of solubilized starch were used to calculate the swelling power and solubility percentage, respectively.

(1) $$\mathrm{Solubility}\;(\%)\;=\;\frac{Dry\;weight\;\mathrm{of}\;solubilized\;starch}{Dry\;weight\;of\;starch}\times 100$$

(2) $$\mathrm{Swelling}\;(\%)\;=\;\frac{Wet\;weight\;of\;swollen\;starch\;sediments}{Dry\;weight\;of\;starch\;x\;(100\;-\;\%\;solubility)}\times 100$$

#### 2.3.4. Moisture Content

The moisture content of all samples was evaluated using a Sartorius MA-50 moisture analyzer (Goettingen, Germany). Approximately 1 g of sample was accurately weighed on to a sample pan and the heating cycle was then started. The temperature was brought up to 105 °C, where it remained until a constant weight of the sample was obtained. The moisture content percentage was calculated by the weight loss of the sample due to heating. The test was repeated in triplicate.

#### 2.3.5. Scanning Electron Micrograph (SEM)

The morphological characteristics of samples were investigated with a Philips XL 30 ESEM instrument (FEI Company, Hillsboro, OR, USA). The sample powder was fixed on an aluminum stub and sputter-coated with a 30–50 nm layer of gold. All samples were then examined using the acceleration voltage at 10–20 kV under low vacuum mode (0.7–0.8 Torr).

#### 2.3.6. Powder Characteristics

The particle size and particle size distribution of samples were determined using a Malvern Mastersizer S (Malvern Instrument, Malvern, UK). The sample was dispersed in methanol for the measurement. The values D10, D50, D90, and mean diameter of the particle size distribution were recorded. True density (ρ_true_) was determined using an Accupyc II 1340 pycnometer (Micromeritics, Norcross, GA, USA). A sample, pre-dried at 60 °C for 24 h, was placed in the sample cup, followed by purging with helium gas at 25–30 °C for 10 times. True density was calculated using the gas displacement method.

### 2.4. Pharmaceutical Properties Evaluation

#### 2.4.1. Flow Property

Flow property was evaluated via determination of an angle of repose (AR) and compressibility index (CI) on the freshly spray-dried samples (with moisture content between 4%–5%) to minimize the effect of moisture content on flowability. AR was manually measured using a fixed funnel method [21]. A cone was formed by pouring the sample powder through a funnel. The height of the cone and the radius of the base of the cone were measured, and the angle of repose was calculated by the equation;

(3) $$\theta ={tan}^{-1}\;(\frac{h}{r})$$

where θ is the angle of repose (°), *h* is the height of the sample cone, and *r* is the radius of the base of the sample cone.

Bulk density was calculated from the bulk volume, obtained by measuring the volume of 20 g sample powder in a 50 mL graduated cylinder. The cylinder was then mechanically tapped using a Jolting volumeter (Stav 2003, Erweka, Langen, Germany). 500 times to obtain the tapped volume used to calculate the tapped density. All tests were repeated in triplicate. CI was calculated from the bulk and tapped densities using the equation

(4) $$\%\;\mathrm{CI}=\left(\frac{Tapped\;density\;-\;Bulk\;density\;}{Tapped\;density}\right)\times 100$$

#### 2.4.2. Compression Behavior

##### Tablet Preparation

A flat-faced punch with an 8.4 mm diameter was used to produce the tablets. The punch surface was pre-lubricated with magnesium stearate suspension before compression. The sample powder was compressed with the compression pressure of 49, 98, 147, and 196 MPa, using a hydraulic press machine (C, Carver, Wabash, IN, USA). Each tablet was accurately weighed, with a diameter (mm) and thickness (mm) measured using a digital Vernier caliper. The data were used to analyze the compression behavior.

##### Tablet Tensile Strength and Porosity

The tablet breaking force of the compacts was determined using a PTB-311 tablet breaking force tester (Pharmatest, Hainburg, Germany). The tablet tensile strength can then be calculated using Fell and Newton’s method [22],

(5) $$\sigma_{x}=\frac{2X}{dt}$$

where σ_x_ is the tensile strength (MPa), x is the hardness (N), d is the diameter of the compact (mm), and t is the thickness of the compact (mm).

The solid fraction (SF) and tablet porosity (ε) were evaluated based on tablet weight (Wt), true density (ρ_true_), and tablet volume (V) using the following equations [11];

(6) $$\mathrm{SF}=\frac{Wt}{\rho_{\mathrm{true}}\;x\;V}$$

(7) ε = 1 − SF

##### Plastic Deformation Property

Plastic deformation property was determined using Heckel analysis. This model assumed that powder volume reduction during powder compression followed first-order kinetic. Plastic deformation property was analyzed from the linear part of the plot as in the following equations [23].

(8) $$D=\;\frac{P_{A}}{P_{T}}$$

(9) $$\ln\frac{1}{1-D}=kP+A$$

where D is the relative density of the compact at the applied pressure *P*, *P_A_* is the apparent density of the compact at pressure *P*, *P_T_* is the true density of the sample powder. *k* parameter represents the constant dependent on the material, while yield pressure (Py) is inversely related to the ability of the material to deform plastically under pressure. *P* is the applied pressure, and *A* expresses as the initial particle rearrangement and the initial volume of the compact during die filling.

Powder compressibility can be determined with Kawakita analysis that was developed to study the powder consolidation using the degree of volume reduction with applied pressure [24],

(10) $$C=1\;-\;\frac{P_{0}}{P_{A}}$$

(11) $$\frac{P}{C}=\frac{P}{a}+\frac{1}{ab}$$

where *C* is volume of the powder during compression, *P_0_* is the bulk density of the sample, *P_A_* is the apparent density of the compact at pressure *P*, *P/C* is a plot against *P* to obtain values of *a* and *b*. A constant *a* value represents the total volume reduction of the powder bed, while *b* value indicates the yield strength of the material. 

#### 2.4.3. Disintegration Property

Tablets of pure excipients were prepared using a hydraulic press machine at 98 MPa compression pressure. The disintegration test was conducted on six tablets according to the standard USP method [25], using a basket apparatus. The disintegration of the tablet was observed, and the time of recording.

### 2.5. Statistics

All tests were carried out at least in triplicate. SPSS (version 19.0) was employed to carry out one-way analysis of variance (ANOVA). Statistical significance tests were performed using Tukey’s honestly significant difference (HSD) multiple range test at a 95% confidence level (*p* <0.05).

## 3. Results and Discussion

### 3.1. Selection of an Optimal Spray Drying Condition and Preparation of CSs

**Figure 1:**
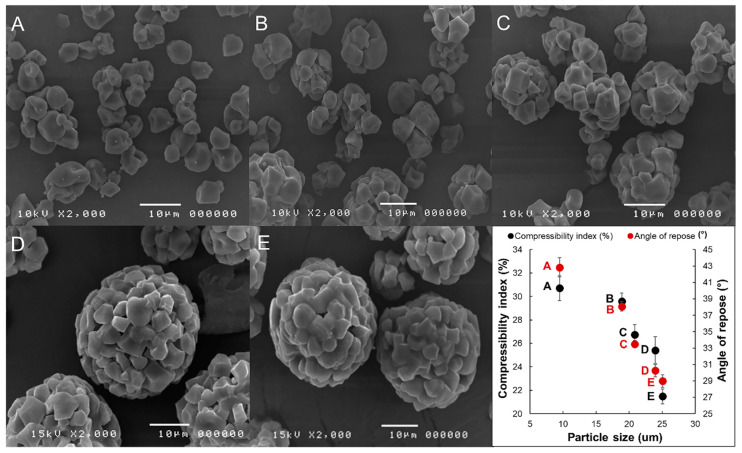
SEM images, particle mean diameter, and flowability parameters of preliminary co-processed rice starches (PCSs) prepared using different spray drying condiditons (nozzle tip size, solid content level). (**A**) 0.7 mm, 20%; (**B**) 1.4 mm, 20%; (**C**) 2.0 mm, 20%; (**D**) 2.0 mm, 30%; and (**E**) 2.0 mm, 40%.

The optimal spray drying condition was selected based on the particle characteristics of PCSs, including particle morphology, particle size distribution, and flowability. The SEM images of PCSs revealed that the shape and size of the co-processed particles were highly influenced by spray nozzle tips and solid content (Figure 1A–D). As the size of the nozzle tip was increased, the shape of PCSs became more spherical. The adsorption of silicon dioxide on the co-processed particles was more evident in PCSs with small particle sizes. The co-processed starch with higher solid contents provided more agglomerate particles. Moreover, the particle size of PCSs was considerably increased with the size of the nozzle tip and the solid content. These results were supported by the flowability study. The PCS prepared with the largest nozzle tip size, and solid content (Figure 1E) showed the best flow property. It was classified as “excellent” on the AR rating scale, and also exhibited the largest particle size. At above 40% solid content, the co-processed suspension was extremely viscous, and the spray drying was not possible, even with the use of a 2.0 mm nozzle tip, which was the largest available size for a B-290 mini spray dry machine. Therefore, the nozzle tip with a 2.0 mm pore size and 40% solid content was selected as the optimal condition for CSs preparation. The effect of silicification levels and CCMS concentrations on CSs properties were subsequently investigated.

### 3.2. FT-IR

**Figure 2:**
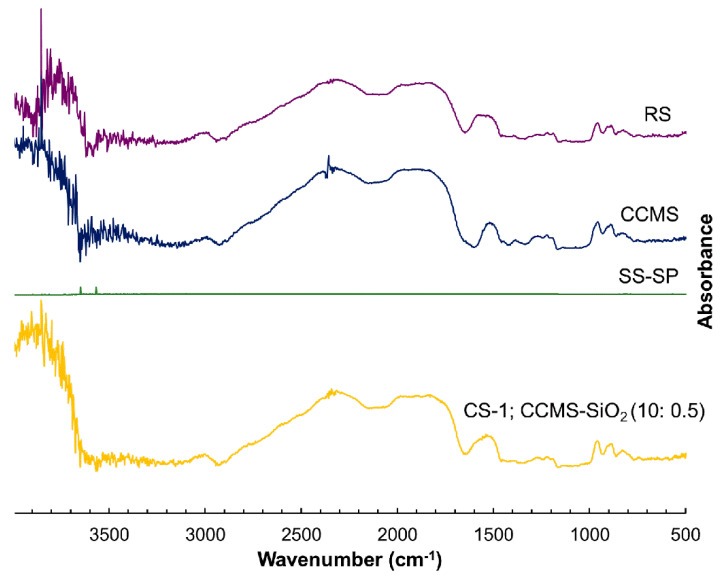
FT-IR spectra of native rice starch (RS), cross-linked carboxymethyl rice starch (CCMS), spray-dried silicon dioxide (SS-SP), and a co-processed rice starch-based excipient (CS-1; CCMS:SiO_2_, 10:0.5).

The carbonyl (C=O) peak of the carboxylate (COO^−^) group was observed at 1600 cm^−1^ of CCMS, confirming the substitution of the hydroxyl group with the carboxymethyl groups (Figure 2) [26]. O-H stretching at 1648 cm^−1^ was detected in the spectra of RS and CS-CCMS:SiO_2_ (10:0.5) [27]. The OH broad band between 3600–3000 cm^−1^ and C-H stretching at about 2900 cm^−1^ were observed in RS, CCMS, and CS-CCMS:SiO_2_ (10:0.5). The band appeared at 1033 cm^−1^ could be assigned to the vibration of Na_2_O·SiO_2_ to Na_2_O·3SiO_2_ that was shown in SS-SP [28]. The FT-IR spectra of co-processed materials showed no additional peak, nor any shift in the peak position compared to that of the parent excipients, indicating that no chemical interaction took place during the co-processing.

### 3.3. XRD

**Figure 3:**
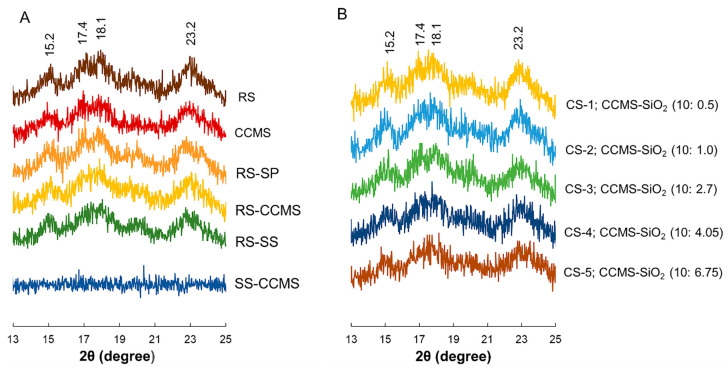
X-ray diffraction pattern of (**A**) control samples, and (**B**) co-processed rice starch-based samples (CSs) with 10% CCMS and different SiO_2_ levels (0.5%–6.75%).

RS, RS-SP, RS-CCMS, and RS-SS displayed an A-type XRD pattern with strong peaks at 15.2, 17.4, 18.1, and 23.2°, respectively (Figure 3A) [4]. The peak intensity of CCMS was slightly lower than that of RS, which corresponded to a loss of crystallinity on starch molecules [29]. This effect was not significantly observed in the XRD pattern of RS-CCMS, indicating that CCMS concentration used in the formulation did not impact the crystallinity of starch. On the other hand, silicon dioxide co-processing reduced the structural crystallinity of CSs (Figure 3B). This was clearly observed as decreased peak intensity in XRD patterns of CSs with high silicification levels (4.05%–6.75%). The presence of silicon dioxide provided an alkaline environment which promoted the gelatinization of starch molecules, leading to increases of the amorphous region [30]. The change in the degree of crystallinity could affect the physicochemical and pharmaceutical properties of materials, such as solubility, swellability, and compressibility [31].

### 3.4. Solubility, Swelling Property, and pH

Native (RS) and spray dried (RS-SP) rice starches exhibited low water solubility and limited swellability. The co-processing with silicon dioxide and CCMS significantly improved starch solubility and swellability, respectively (Table 1). As a result, most CSs exhibited superior solubility and swellability compared to RS and RS-SP. RS-SS showed greater solubility than RS due to the hygroscopic behavior of silicon dioxide [32]. The solubility of CSs was gradually improved with the increase in silicification levels. Higher silicification levels promoted a more alkaline environment, thus increased starch gelatinization and solubility [30]. On the other hand, the presence of CCMS in the co-processed particles improved starch swellability due to the hydrophilic carboxymethyl group on CCMS molecules. This functional group enhanced water penetration into the co-processed particles [33], as evidenced by a considerably greater value for RS-CCMS swellability than those for RS, RS-SP, and RS-SS. The swellability of CSs was slightly affected by the CCMS levels. The CSs containing 15% CCMS showed the highest swellability, although the difference from other samples was not significant. The pH of the starch solution was also affected by the silicification levels. A silicon dioxide amount higher than 4% provided alkaline pH of starch solution, while below this percentage, the samples showed a neutral pH solution. This result related to the solubility of CSs, as the increase of pH solution, promoted the gelatinization of starch granules.

### 3.5. Moisture Content

The moisture content of RS and RS-SP complied with the USP moisture content specification of not more than 15% (Table 2) [34]. CSs showed moisture content in the range of 5.45% to 8.25%, suggesting that silicification had no effect on CSs moisture content. Prosolv^®^ and Tablettose^®^ moisture contents were within the specification [35,36].

### 3.6. Scanning Electron Micrograph (SEM)

**Figure 4:**
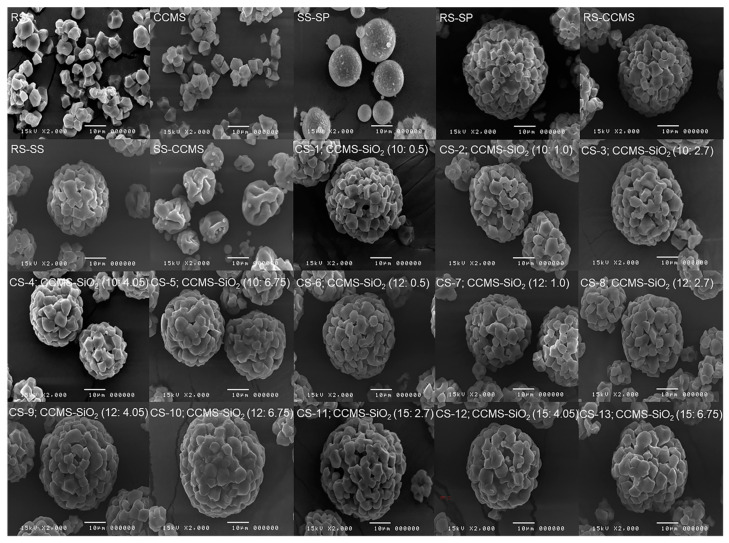
SEM images of RS, CCMS, SS-SP, RS-SP, RS-CCMS, RS-SS, SS-CCMS, and CSs with different CCMS (10%, 12%, 15%) and SiO_2_ (0.5%, 1.0%, 2.7%, 4.05%, 6.75%) levels.

SEM images of RS, CCMS, and SS-CCMS showed particles with irregular, polyhedral shape (Figure 4), while the shapes of all CSs particles were spherical agglomerates with a smooth surface. The small particles of silicon dioxide were seen adsorbed on the surface of the co-processed particles. As silicification levels increased, starch granules started to enlarge, leading to a slight increase in the granule size and agglomeration of starch granules. This effect was clearly observed in high concentrations of CCMS. The adsorption of silicon dioxide on the co-processed particle was remarkably observed in SS-CCMS, which exhibited small particle size.

### 3.7. Powder Characteristics (Particle Size, and Powder Density)

**Figure 5:**
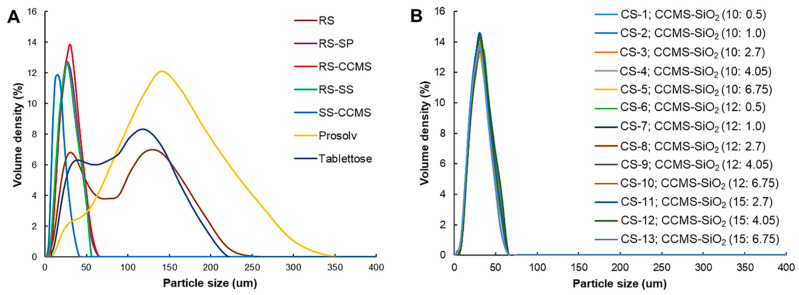
Particle size distribution curves of (**A**) RS, RS-SP, RS-CCMS, RS-SS, SS-CCMS, and commercial DC excipients, and (**B**) CSs with different CCMS (10%, 12%, 15%) and SiO_2_ (0.5%, 1.0%, 2.7%, 4.05%, 6.75%) levels.

The spray drying technique afforded the particles with a narrow size distribution that can be observed from a narrow unimodal particle size distribution curve of the RS-SP, and CSs (Figure 5). RS-CCMS, RS-SS, and CSs had very similar particle size and particle size distribution. The particle sizes of CSs were in the range of 24.33–27.03 um, which were smaller than that of RS, Prosolv^®^, and Tablettose^®^ (Table 2). Prosolv^®^ exhibited the largest particle size with a wide size distribution (Figure 5A). The particle size of RS and Tablettose^®^ were similar, and both showed a biomodal particle size distribution curve. The size and shape of particles had a direct impact on the pharmaceutical properties of materials. Prosolv^®^ and Tablettose^®^ exhibited excellent flowability due to their large particle size, while improved flowability of CSs was mainly attributed to a combination of the increased size and spherical shape created by the use of spray drying technique [37]. CSs showed the narrowest particle size distribution (Figure 5B), which could reduce tableting problems such as segregation and agglomeration during the blending step [38].

The bulk, tapped, and true densities (Table 2) showed that RS had the lowest density values compared with that of RS-SP, and other co-processed materials. Compared with RS-SP alone, this co-processing affected the densities of co-processed materials that were progressively increased with increases in the silicification level. SS-CCMS showed the highest density values as it contained the largest amount of silicon dioxide. This finding was in agreement with previous studies on cellulose II-SiO_2_ and Chitin-SiO_2_ composites [18,39]. Bulk and tapped densities of Prosolv^®^ were lower than those of co-processed starches, while Tablettose^®^ showed higher bulk and tapped densities than those of CSs. The increase of bulk density facilitated the tableting process, especially in the die-filling process [40], whereas powder, which exhibited very low bulk density may need densification before tableting [41].

### 3.8. Flow Property

**Figure 6:**
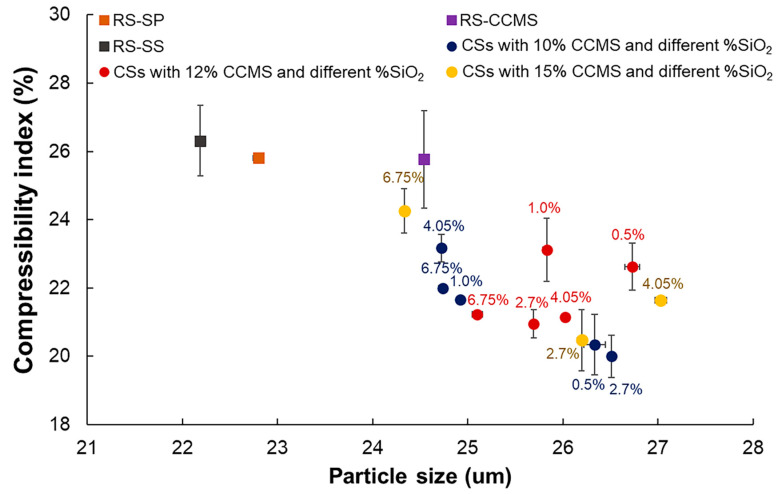
Relationship between the particle size of CSs with different CCMS (10%, 12%, 15%) and SiO_2_ (0.5%, 1.0%, 2.7%, 4.05%, 6.75%) levels and the flowability (CI, %).

The spray drying technique altered the morphology of the co-processed particles in such a way that it enhanced the flowability of materials. The particles with irregular shape (i.e., RS) exhibited poor flow character (AR = 40.0 ± 1.1; CI = 29.3 ± 0.4), while agglomerate spherical particles (i.e., RS-SP, RS-SS, and RS-CCMS) showed superior flowability [42]. SS-CCMS exhibited the worst flow character (AR = 43.2 ± 1.0; CI = 62.5 ± 1.0, data not shown on graph), which could be due to the particle shape and over addition of silicon dioxide that inhibited flowability [43]. All CSs showed better flowability than RS and the control samples. CI values of the CSs were in the range of 20.0–24.3 (fair to passable). AR values, between 28.0–31.2, were classified as excellent flow character. The results suggested that the improvement in flowability of CSs was dependent on the spray drying technique rather than the silicification and CCMS levels. The CSs with 2.7% of silicon dioxide showed slightly superior flowability to other CSs, and comparable to that of commercial DC excipients (Figure 6). The concentration of CCMS showed no significant effect on particle flowability as it did not impact the size and shape of CSs particles. Compared to the commercial DC excipient, Tablettose^®^ showed excellent flowability due to the spray drying technique that caused particle agglomeration [44]. Flowability of Prosolv^®^ was improved by the coating of silicon dioxide on the particle surface, and these effects were also observed in the flowability of CSs [45].

### 3.9. Compression Behavior

#### 3.9.1. Tensile Strength

**Figure 7:**
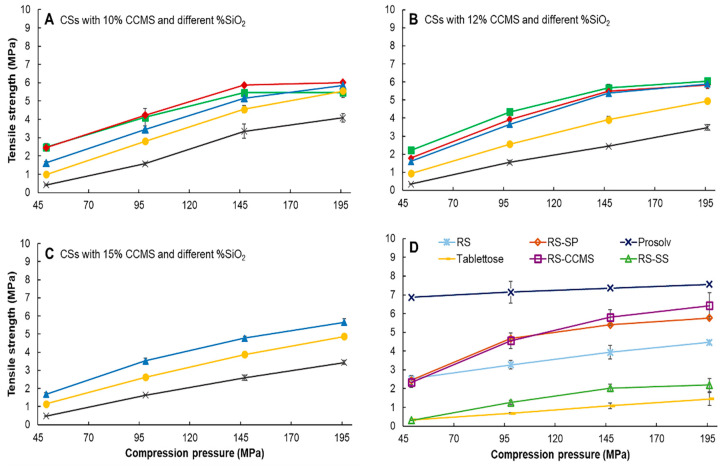
Tablet tensile strength of CSs with different CCMS and SiO_2_ levels. (**A**) CSs with 10% CCMS, (**B**) CSs with 12% CCMS, (**C**) CSs with 15% CCMS. and (**D**) control samples. SiO_2_ levels were represented as (■) 0.5%, (♦) 1.0%, (▲) 2.7%, (●) 4.05%, and (x) 6.75%.

RS exhibited good compressibility owing to the “interlock” behavior of particles with small particle size and irregular shape. The CSs (Figure 7A–C) and RS-SP tablets (Figure 7D) showed improvement in compressibility, which was superior to that of RS. The co-processing with CCMS (RS-CCMS) improved the compressibility of the material as the crystallinity was decreased and plastic deformation was promoted [46]. Thus, the increase of CCMS levels slightly improved the tablet tensile strength. On the other hand, silicon dioxide co-processing inhibited particle binding that can be observed in the compressibility profile of CSs with high silicification levels, RS-SS; this could be a result of the antiplasticization of silicon dioxide that incorporated into the core of co-processed particles. Prosolv^®^, a microcrystalline cellulose-based excipient, exhibited the best tensile strength due to the plasticity of MCC that created a continued matrix during compaction [12], whereas the tensile strength of Tablettose^®^ was the worst compared to the other excipients due to the brittle characteristic of lactose [47].

#### 3.9.2. Plastic Deformation Property

Heckel analysis was the most widely used technique to study the plastic deformation property of a material by way of calculating the Heckel yield pressure (Py) [48]. The lower Py value indicated the greater amount of plasticity of the material. Prosolv^®^ showed a low Py value (156.25), implying a high degree of plastic deformation and compressibility of material (Table 3). In contrast, a brittle material such as Tablettose^®^ yielded a high Py value (243.90), meaning a large amount of energy was required to break the particle into small fragments during the compression process [49]. As a result, Tablettose^®^ was difficult to compress into a tablet [50]. The Py values of RS, RS-SP, and co-processed starches were in between the two commercial DC excipients as RS inherently exhibited plastic deformation with limited fragmentation [6]. RS-CCMS showed a lower Py value than that of RS, as the addition of CCMS caused a reduction in the crystallinity of starch. In contrast, the presence of silicon dioxide elevated the Py value of starch, as seen in RS-SS. This result suggested that silicon dioxide enhanced the brittle characteristic of materials [45]. Py values of CSs were in the range of 151.52 to 204.08, which were affected by the crystallinity of the CSs. The CSs with the higher amorphous region, as exhibited by the low peak intensity in the XRD pattern, showed lower Py values, which indicated more plastic deformation.

The constant *a* and 1/*b* values from the Kawakita equation were used to determine the powder behavior of the materials (Table 3). Prosolv^®^ had the highest *a* value, which indicated the highest compressibility [51]. In contrast, Tablettose^®^, which mainly consolidated through fragmentation mechanism, showed low volume reduction during compaction [52]. The co-processing with silicon dioxide decreased the *a* value of CSs. The effect was more noticeable in high silicification levels. These results related to the tensile strength of the co-processed starch. At 0.5%–2.7% of silicon dioxide, the tensile strength of CSs was not affected by silicification levels. At above 2.7%, the tensile strength of CSs decreased proportionally to the minimum at 6.75% of silicon dioxide. The 1/*b* value related to the compression pressure required to achieve one half of the total volume reduction, which indicated the plasticity of material [50]. The results of 1/*b* value of all excipients were in accordance with the Py values from the Heckel plot. Prosolv^®^ showed a high 1/*b* value, while that of Tablettose^®^ was the lowest compared to other excipients.

Based on the results from Heckel and Kawakita analyses, the improvement in the plasticity of CSs could be due to the increase of the amorphous region as confirmed by the XRD pattern of CSs. An amorphous material exhibited a liquid-like structure that promoted molecular mobility of material. Therefore, high amorphous content facilitated plastic deformation and compressibility of excipient [46]. However, the CSs with low Py values showed low tablet tensile strength, likely due to the antiplasticization phenomena. Small particles of silicon dioxide incorporated into the core of co-processed particles may act as an antiplasticizer that could elevate glass transition (Tg) of the material. At a temperature below Tg, amorphous materials normally exhibited hard and brittle characteristics similar to the crystalline material that impeded compressibility [53].

### 3.10. Disintegration Property

**Figure 8:**
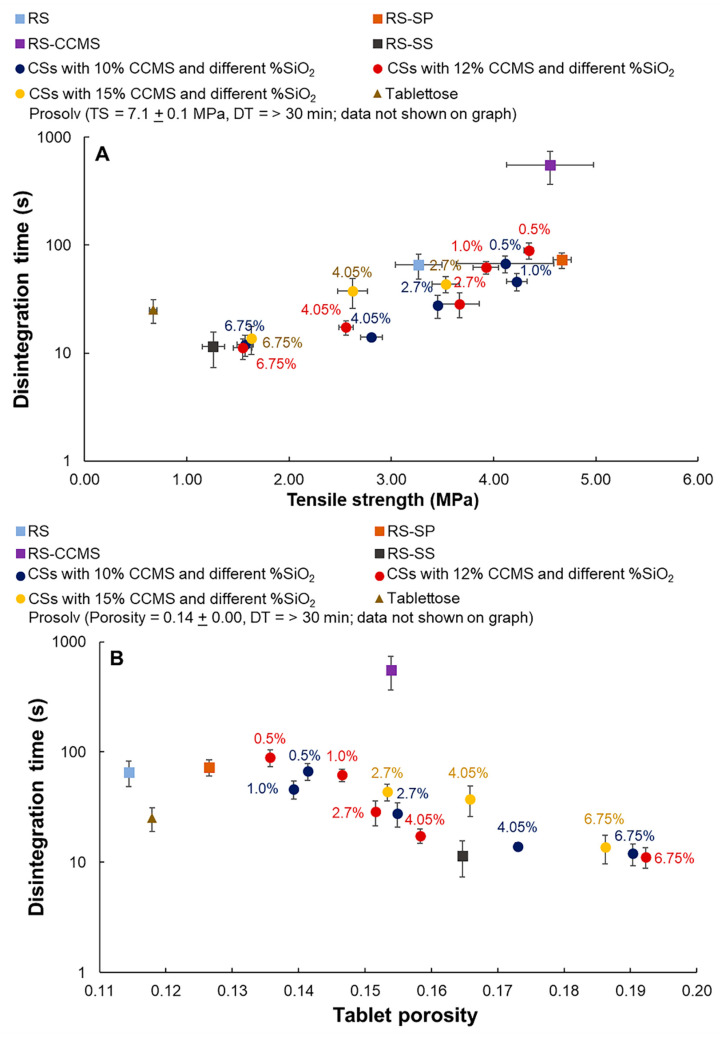
Effects of tablet tensile strength (**A**) and tablet porosity (**B**) on the disintegration time of rice starch and co-processed rice starch tablets.

RS and RS-SP tablets showed modest disintegration times at 65.7 ± 17.1 and 73.0 ± 12.7 s, respectively. The CSs tablets showed varied disintegration times between 11.2 and 89.3 s. The longest and shortest disintegration times among tested samples were observed from RS-CCMS (553.4 ± 188.1 s) and RS-SS (11.5 ± 4.2 s) tablets, respectively, which suggested that CCMS and silicon dioxide played significant roles in the disintegration process. CCMS normally adsorbed water and swelled to perform as a superdisintegrant when used at a low concentration [33]. However, at higher concentrations, it formed a viscous gel that retarded the penetration of water into the tablets, thus increasing the disintegration time, as observed in RS-CCMS and CSs tablets with higher CCMS concentrations. In contrast, the presence of silicon dioxide shortened the disintegration time, primarily due to its high water adsorption capacity [54]. The results showed that the disintegration time of CSs tablets increased moderately in those with increased CCMS concentrations, while the increase in silicification levels gradually exhibited the opposite effect. In addition, the effects of tablet tensile strength and porosity on the disintegration of CSs tablets must be taken into account. A high tensile strength often correlated with a higher tablet density, which made it more difficult for water to penetrate into the tablets, thus slowing down the disintegration process (Figure 8A). On the contrary, a tablet with higher porosity tended to disintegrate faster as water can easier migrate into the tablets and cause the break up (Figure 8B) [55]. Figure 8 shows that silicon dioxide contributed significantly to the tensile strength and porosity of CSs tablets. The tablets with higher silicification levels showed lower tablet tensile strength and higher tablet porosity. Comparison of tablets with similar porosity revealed that the concentration of CCMS in the formulation affected the disintegration time and that co-processed starch CS-3 (CCMS:SiO_2_ 10:2.7) exhibited the best characteristics. Its disintegration time was comparable to that Tablettose^®^ tablet. The long disintegration time of Prosolv^®^ tablets (>n30 min) was likely a result of a low disintegration efficacy of microcrystalline cellulose and a high tablet tensile strength due to the formation of strong intraparticular bonds [56].

## 4. Conclusions

A new co-processed, rice starch-based excipient (CS), prepared by spray drying a mixture of rice starch, CCMS and sodium silicate, exhibited good flowability, compressibility, swellability, and disintegration properties. The FT-IR spectra showed no change or shift in the peak position, which confirmed that only physical modification took place during the preparation process. The silicification increased gelatinization and solubility of the CSs, while CCMS affected swellability, in a concentration-related fashion. The results were supported by the XRD pattern, which showed the loss of amorphous region in high silicified samples. In addition, the powder densities were enhanced with silicification levels. The particle size and SEM images revealed that the particle morphology was mainly impacted by the spray drying condition. The larger spray nozzle tip with higher solid content provided more agglomerate spherical shape that promoted the flowability of the co-processed starches. The incorporation of CCMS added disintegration property, while it also enhanced the tensile strength of tablets. Silicon dioxide contributed to the porosity of the powder and affected disintegration time. The best co-processed rice starch, CS-3 (CCMS:SiO_2_ 10:2.7), showed a good tensile strength that lay between that of Prosolv^®^ (plastic material) and Tablettose^®^ (brittle material). It also showed a rapid disintegration time, which was superior to that of the Prosolv^®^ tablet and was comparable to the Tablettose^®^ tablet. Therefore, the CSs could potentially be applied as a multifunctional excipient (free flowing filler-binder and disintegrant) for direct compression of tablets.

## Figures and Tables

**Table 1:** Co-processed conditions, solubility, swellability, and pH of native rice starch (RS), spray-dried rice starch (RS-SP), and co-processed starches.

Samples	Solubility (%) n = 3	Swellability (%) n = 3	pH n = 3
CS-1; CCMS-SiO_2_ (10:0.5)	0.86 ± 0.16 ^a^	5.80 ± 0.41 ^b,c^	7.0 ± 0.0 ^a^
CS-2; CCMS-SiO_2_ (10:1.0)	1.86 ± 0.61 ^a^	5.57 ± 0.71 ^b,c^	7.1 ± 0.3 ^a^
CS-3; CCMS-SiO_2_ (10:2.7)	3.39 ± 0.42 ^a,b^	5.37 ± 0.71 ^b,c^	7.2 ± 0.1 ^a^
CS-4; CCMS-SiO_2_ (10:4.05)	5.14 ± 0.12 ^b,c^	5.32 ± 0.38 ^b,c^	10.1 ± 0.1 ^b^
CS-5; CCMS-SiO_2_ (10:6.75)	7.70 ± 0.64 ^c^	5.35 ± 0.52 ^b,c^	10.0 ± 0.1 ^b^
CS-6; CCMS-SiO_2_ (12:0.5)	2.28 ± 0.09 ^a,b^	5.14 ± 0.29 ^b,c^	7.2 ± 0.3 ^a^
CS-7; CCMS-SiO_2_ (12:1.0)	2.83 ± 0.12 ^a,b^	5.32 ± 0.38 ^b,c^	7.0 ± 0.0 ^a^
CS-8; CCMS-SiO_2_ (12:2.7)	4.90 ± 0.46 ^b,c^	5.24 ± 0.32 ^b,c^	7.1 ± 0.1 ^a^
CS-9; CCMS-SiO_2_ (12:4.05)	5.61 ± 0.29 ^b,c^	5.51 ± 0.11 ^b,c^	10.0 ± 0.1 ^b^
CS-10; CCMS-SiO_2_ (12:6.75)	8.08 ± 0.31 ^c^	5.24 ± 0.08 ^b,c^	10.1 ± 0.1 ^b^
CS-11; CCMS-SiO_2_ (15:2.7)	4.30 ± 0.18 ^b^	6.33 ± 0.34 ^c^	7.0 ± 0.1 ^a^
CS-12; CCMS-SiO_2_ (15:4.05)	5.66 ± 0.16 ^b,c^	5.67 ± 0.38 ^b,c^	10.0 ± 0.1 ^b^
CS-13; CCMS-SiO_2_ (15:6.75)	8.12 ± 0.15 ^c^	6.01 ± 0.37 ^b,c^	10.1 ± 0.0 ^b^
RS	1.12 ± 0.41 ^a^	3.49 ± 1.23 ^a^	7.4 ± 0.2 ^a^
RS-SP	1.26 ± 0.25 ^a^	3.13 ± 0.20 ^a^	7.2 ± 0.1 ^a^
RS-CCMS	1.53 ± 0.12 ^a^	4.97 ± 0.58 ^b^	7.0 ± 0.1 ^a^
RS-SS	4.36 ± 0.19 ^b^	3.73 ± 0.11 ^a^	7.0 ± 0.0 ^a^
SS-CCMS	35.26 ± 0.36 ^d^	14.89 ± 0.42 ^d^	10.0 ± 0.0 ^b^

Data are displayed as Mean ± Standard Deviation. Means followed by a common letter (a-d) are not significantly different by Tukey HSD test at the 5% level of significance (*p* <0.05).

**Table 2:** Density, moisture content, and particle size of CSs, RS, RS-SP, and commercial DC excipients.

Materials	Density			Moisture Content n = 3	Particle Size (um) n = 3
	Bulk (n = 3)	Tapped (n = 3)	True (n = 3)		
CS-1; CCMS-SiO_2_ (10:0.5)	0.4 ± 0.0 ^c^	0.6 ± 0.0 ^c^	1.5320 ± 0.0004 ^b^	7.61 ± 0.12 ^e^	26.33 ± 0.11 ^e^
CS-2; CCMS-SiO_2_ (10:1.0)	0.4 ± 0.0 ^c^	0.6 ± 0.0 ^c^	1.5383 ± 0.0003 ^c^	7.13 ± 0.39 ^d,e^	24.92 ± 0.02 ^c^
CS-3; CCMS-SiO_2_ (10:2.7)	0.5 ± 0.0 ^d^	0.6 ± 0.0 ^d^	1.5598 ± 0.0004 ^d^	6.91 ± 0.10 ^d,e^	26.51 ± 0.03 ^e,f^
CS-4; CCMS-SiO_2_ (10:4.05)	0.5 ± 0.0 ^d^	0.6 ± 0.0 ^e^	1.5749 ± 0.0002 ^e^	7.19 ± 0.54 ^d,e^	24.72 ± 0.03 ^c^
CS-5; CCMS-SiO_2_ (10:6.75)	0.5 ± 0.0 ^e^	0.7 ± 0.0 ^f^	1.5901 ± 0.0003 ^f^	5.45 ± 0.14 ^c^	24.73 ± 0.04 ^c^
CS-6; CCMS-SiO_2_ (12:0.5)	0.4 ± 0.0 ^c^	0.6 ± 0.0 ^c^	1.5279 ± 0.0002 ^b^	6.16 ± 0.44 ^c,d^	26.73 ± 0.08 ^f^
CS-7; CCMS-SiO_2_ (12:1.0)	0.4 ± 0.0 ^c^	0.6 ± 0.0 ^c^	1.5334 ± 0.0004 ^b^	6.69 ± 0.26 ^d^	25.83 ± 0.04 ^d^
CS-8; CCMS-SiO_2_ (12:2.7)	0.5 ± 0.0 ^d^	0.6 ± 0.0 ^d^	1.5572 ± 0.0002 ^d^	7.13 ± 0.10 ^d,e^	25.69 ± 0.02 ^d^
CS-9; CCMS-SiO_2_ (12:4.05)	0.5 ± 0.0 ^e^	0.7 ± 0.0 ^e,f^	1.5690 ± 0.0003 ^e^	6.99 ± 0.13 ^d,e^	26.02 ± 0.03 ^d,e^
CS-10; CCMS-SiO_2_ (12:6.75)	0.5 ± 0.0 ^e^	0.7 ± 0.0 ^f^	1.5888 ± 0.0009 ^f^	6.88 ± 0.21 ^d,e^	25.10 ± 0.05 ^c^
CS-11; CCMS-SiO_2_ (15:2.7)	0.50.0 ^e^	0.6 ± 0.0 ^d^	1.5589 ± 0.0004 ^d^	8.25 ± 0.23 ^e^	26.20 ± 0.05 ^d,e^
CS-12; CCMS-SiO_2_ (15:4.05)	0.5 ± 0.0 ^d^	0.6 ± 0.0 ^e^	1.5716 ± 0.0003 ^e^	6.88 ± 0.38 ^d,e^	27.03 ± 0.06 ^f^
CS-13; CCMS-SiO_2_ (15:6.75)	0.5 ± 0.0 ^d^	0.7 ± 0.0 ^f^	1.5928 ± 0.0005 ^f^	5.78 ± 0.23 ^c^	24.33 ± 0.01 ^c^
RS	0.3 ± 0.0 ^a^	0.5 ± 0.0 ^b^	1.5291 ± 0.0009 ^b^	10.75 ± 0.51 ^f^	60.42 ± 0.06 ^g^
RS-SP	0.4 ± 0.0 ^c^	0.6 ± 0.0 ^c,d^	1.5154 ± 0.0003 ^a^	5.77 ± 0.29 ^c^	22.80 ± 0.06 ^b^
RS-CCMS	0.4 ± 0.0 ^b^	0.5 ± 0.0 ^c^	1.5225 ± 0.0008 ^a^	6.24 ± 0.14 ^c,d^	24.54 ± 0.03 ^c^
RS-SS	0.5 ± 0.0 ^c,d^	0.6 ± 0.0 ^d,e^	1.5466 ± 0.0004 ^c^	5.28 ± 0.14 ^c^	22.19 ± 0.02 ^b^
SS-CCMS	0.6 ± 0.0 ^f^	1.5 ± 0.0 ^h^	1.7553 ± 0.0007 ^h^	3.11 ± 0.09 ^b^	13.55 ± 0.03 ^a^
Prosolv^®^	0.3 ± 0.0 ^a^	0.4 ± 0.0 ^a^	1.6023 ± 0.0009 ^g^	4.97 ± 0.24 ^c^	110.88 ± 0.50 ^i^
Tablettose^®^	0.6 ± 0.0 ^f^	0.7 ± 0.0 ^g^	1.5464 ± 0.0034 ^c^	0.46 ± 0.05 ^a^	66.49 ± 0.15 ^h^

Data are displayed as Mean ± Standard Deviation. Means followed by a common letter (a-i) are not significantly different by Tukey HSD test at the 5% level of significance (*p* < 0.05).

**Table 3:** Heckel and Kawakita calculated constants of CSs, RS, RS-SP, RS-CCMS, RS-SS, SS-CCMS, and commercial DC excipients.

Samples	Heckel Constants			Kawakita Constants		
	Py	A	r^2^	a	1/b	r^2^
CS-1; CCMS-SiO_2_ (10:0.5)	200.00	1.3254	0.9342	0.69	4.86	0.9999
CS-2; CCMS-SiO_2_ (10:1.0)	204.08	1.4015	0.9622	0.69	3.94	0.9999
CS-3; CCMS-SiO_2_ (10:2.7)	188.68	1.2377	0.8898	0.68	6.66	0.9999
CS-4; CCMS-SiO_2_ (10:4.05)	175.44	1.1102	0.8999	0.68	8.74	0.9997
CS-5; CCMS-SiO_2_ (10:6.75)	151.52	0.9522	0.9640	0.66	13.91	0.9997
CS-6; CCMS-SiO_2_ (12:0.5)	196.08	1.3628	0.8636	0.70	4.44	0.9999
CS-7; CCMS-SiO_2_ (12:1.0)	196.08	1.3235	0.8774	0.71	5.12	0.9999
CS-8; CCMS-SiO_2_ (12:2.7)	161.29	1.1862	0.9424	0.69	7.31	0.9999
CS-9; CCMS-SiO_2_ (12:4.05)	169.49	1.1640	0.9284	0.66	8.78	0.9999
CS-10; CCMS-SiO_2_ (12:6.75)	161.29	0.9644	0.9569	0.65	13.87	0.9999
CS-11; CCMS-SiO_2_ (15:2.7)	151.52	1.1495	0.9596	0.69	7.45	0.9999
CS-12; CCMS-SiO_2_ (15:4.05)	163.93	1.1099	0.9379	0.67	9.09	0.9999
CS-13; CCMS-SiO_2_ (15:6.75)	163.93	1.0143	0.9677	0.67	11.00	0.9999
RS	196.08	1.5486	0.9519	0.74	5.45	0.9999
RS-SP	161.29	1.3240	0.9044	0.71	5.34	0.9999
RS-CCMS	175.44	1.2507	0.9819	0.74	5.45	0.9999
RS-SS	204.08	1.1767	0.8839	0.69	6.88	0.9999
SS-CCMS	212.77	1.0315	0.9933	0.69	6.88	0.9999
Prosolv^®^	156.25	1.3610	0.9692	0.79	11.39	0.9999
Tablettose^®^	243.90	1.7658	0.9915	0.60	4.16	0.9999

## Abbreviations

AR	angle of repose
CCMS	cross-linked carboxymethyl starch
CI	compressibility Index
CS	co-processed, rice starch-based excipient
DC	direct compression
PCS	preliminary co-processed rice starch
RS	native rice starch
RS-CCMS	rice starch co-processed with CCMS
RS-SP	spray dried rice starch
SS-CCMS	silicon dioxide co-processed with CCMS
SS-SP	spray-dried silicon dioxide
